# Bidirectional Phase Transformations in Multi‐Principal Element Alloys: Mechanisms, Physics, and Mechanical Property Implications

**DOI:** 10.1002/advs.202407283

**Published:** 2024-08-19

**Authors:** Jiayi Sun, Heqing Li, Yujie Chen, Xianghai An

**Affiliations:** ^1^ School of Aerospace Mechanical and Mechatronic Engineering The University of Sydney Sydney NSW 2006 Australia; ^2^ Sydney Nano Institute (Sydney Nano) The University of Sydney Sydney NSW 2006 Australia; ^3^ School of Electrical and Mechanical Engineering The University of Adelaide Adelaide SA 5005 Australia

**Keywords:** bidirectional phase transformation, intrinsic negative stacking fault energy, mechanical properties, Multi‐principal element alloys, phase stability

## Abstract

The emergence of multi‐principal element alloys (MPEAs) heralds a transformative shift in the design of high‐performance alloys. Their ingrained chemical complexities endow them with exceptional mechanical and functional properties, along with unparalleled microscopic plastic mechanisms, sparking widespread research interest within and beyond the metallurgy community. In this overview, a unique yet prevalent mechanistic process in the renowned FeMnCoCrNi‐based MPEAs is focused on: the dynamic bidirectional phase transformation involving the forward transformation from a face‐centered‐cubic (FCC) matrix into a hexagonal‐close‐packed (HCP) phase and the reverse HCP‐to‐FCC transformation. The light is shed on the fundamental physical mechanisms and atomistic pathways of this intriguing dual‐phase transformation. The paramount material parameter of intrinsic negative stacking fault energy in MPEAs and the crucial external factors c, furnishing thermodynamic, and kinetic impetus to trigger bidirectional transformation‐induced plasticity (B‐TRIP) mechanisms， are thorougly devled into. Furthermore, the profound significance of the distinct B‐TRIP behavior in shaping mechanical properties and creating specialized microstructures c to harness superior material characteristics is underscored. Additionally, critical insights are offered into key challenges and future striving directions for comprehensively advancing the B‐TRIP mechanism and the mechanistic design of next‐generation high‐performing MPEAs.

## Graphical abstract

The emergence of multi‐principal element alloys (MPEAs) signifies a transformative breakthrough in high‐performance alloy design. This review explores the unique bidirectional phase transformation in MPEAs, shedding light on fundamental mechanisms, intrinsic and external factors driving this mechanistic process. It underscores its profound significance in shaping mechanical properties and microstructures, offering critical insights into challenges and future directions for advancing MPEAs.

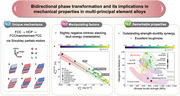

## 1 Introduction

High‐performance metallic materials are indispensable as the essential building blocks for advancing innovations across a wide spectrum of structural applications, spanning next‐generation energy systems, space exploration, transportation networks, and cutting‐edge manufacturing processes.^[^ ^1^ ^]^ As we are facing escalating challenges pertinent to energy efficiency and environmental sustainability, the demands for superb alloys that should be strong, ductile, tough, durable, and reliable have never been more pronounced.^[^ ^2^ ^]^ However, simultaneous attainment of these materials’ characteristics remains a formidable task, given that many of these properties are traditionally viewed as mutually exclusive, which is rooted in the intrinsic nature of dislocation plasticity.^[^ ^3^ ^]^ Consequently, conventional design and fabrication methods often necessitate undesirable compromises in material properties. Over the past few decades, materials scientists have been dedicated to pioneering innovative material design strategies with the goal of achieving a harmonious combination of exceptional properties.^[^ ^4^, ^5^, ^6^, ^7^, ^8^, ^9^ ^]^

Unlike the conventional alloying strategy of incorporating small quantities of secondary atoms into a primary element, a novel approach to alloy design that involves mixing multiple principal elements in considerable concentrations, known as multiple principal element alloys (MPEAs) or high‐entropy alloys (HEAs), has been recently developed.^[^ ^10^, ^11^, ^12^, ^13^ ^]^ The intricate chemical complexities of the MPEAs offer a multitude of opportunities for enhancing material properties due to the vast compositional space that was previously inaccessible.^[^ ^14^, ^15^ ^]^ Extensive investigations have demonstrated that MPEAs not only display an exceptional combination of strength, ductility, and toughness but also present outstanding resistance to fatigue, creep, corrosion, hydrogen embrittlement, thermal fluctuation, and irradiation.^[^ ^13^ ^]^ As a result, MPEAs have ushered in a new era in the development of high‐performing alloys, empowering us to break through to a new level of materials effectiveness that would substantially improve mechanical and energy efficiencies. This advancement will thereby aid in mitigating environmental burdens, promoting sustainability, and underpinning more reliable safety‐critical engineering applications under extreme environments.^[^ ^16^, ^17^, ^18^ ^]^

It has been previously proposed that the elevated configuration entropy stemming from the combination of multiple elements can significantly lower the Gibbs free energy, thereby profoundly stabilizing the single solid solution state.^[^ ^11^, ^12^, ^13^ ^]^ This can facilitate the formation of single‐phase crystal structures including face‐centered cubic (FCC),^[^ ^19^, ^20^, ^21^ ^]^ body‐centered cubic (BCC),^[^ ^22^, ^23^, ^24^ ^]^ and hexagonal close‐packed (HCP) structures^[^ ^25^, ^26^, ^27^ ^]^ within MPEAs. However, from a thermodynamics perspective, configuration entropy is not the sole ingredient influencing the Gibbs free energy. Recent theoretical and experimental investigations have highlighted the crucial roles of formation enthalpy and other entropy contributors in the formation and stabilization of these solid solutions.^[^ ^28^, ^29^, ^30^, ^31^ ^]^ The renowned Cantor alloy (CoCrFeNiMn MPEA) and its derivatives, serving as the prototypes of MPEAs, maintain a stable FCC structure over a wide temperature range, spanning from cryogenic temperatures to alloys’ melting temperatures.^[^ ^19^ ^]^ Their compositional complexities inherent in these alloys promote local fluctuations in composition, chemically heightening the ruggedness of the energy landscape against dislocation plasticity.^[^ ^32^, ^33^, ^34^, ^35^ ^]^ With the advancements demonstrating that the originally strict HEA rule regarding single‐phase stability can be thermodynamically relaxed, mechanistic MPEA design strategies have emerged to induce the twinning‐induced plasticity (TWIP) and transformation‐induced plasticity (TRIP) by elaborately tuning their stacking fault energies (SFEs) through subtle manipulation of chemical compositions.^[^ ^36^, ^37^ ^]^ Such collective inelastic dissipation modes can essentially undertake substantial plasticity, accompanied by the formation of profuse twin/phase boundaries that provide effective barriers for dislocation motion and offer ample room for the storage of additional defects.

Apart from the deliberate alloy design for metastability engineering, thorough theoretical explorations utilizing ab initio calculations have startlingly unveiled the metastable characteristic of the FCC structure in FeMnCrCoNi‐based MPEAs, initially perceived as stable.^[^ ^38^, ^39^, ^40^ ^]^ These studies have also identified a negative SFE in these alloys, indicating that their HCP structure should be more thermodynamically stable due to its lower Gibbs free energy compared to the FCC phase at low temperatures. This implies that the displacive phase transformation from the metastable FCC structure to stable HCP stacking is energetically favorable during plastic deformation. In stark contrast to theoretical implications, experimental investigations have demonstrated abundant deformation twins and very limited phase transformations.^[^ ^38^, ^41^, ^42^, ^43^ ^]^ This highlights the challenge of the FCC‐HCP phase transition being kinetically restricted due to the compositional complexity of MPEAs that confers sophisticated generalized fault energy curve (γ‐surface).^[^ ^41^, ^42^, ^43^, ^44^ ^]^ Therefore, materials scientists are striving to elucidate the competitive or synergistic nature of these microscale displacive plasticity to unravel the nanostructured origins of their superior mechanical properties.

Recently, a novel bidirectional phase transformation has been observed in several MPEAs, involving a sequential phase transformation from the FCC phase to the HCP structure and back to the nanotwinned FCC or FCC structure.^[^ ^45^, ^46^, ^47^, ^48^, ^49^, ^50^, ^51^, ^52^, ^53^ ^]^ It is generally acknowledged that transformations from an unstable or metastable configuration to a stable one are thermodynamically driven by the reduction of Gibbs free energy, which are typically irreversible. Consequently, such unexpected bidirectional mechanistic processes have become a compelling subject of increasing experimental and theoretical investigations within and even beyond the MPEA community.^[^ ^45^, ^46^, ^47^, ^48^, ^49^, ^50^, ^51^, ^52^, ^53^, ^54^, ^55^, ^56^ ^]^ In addition, this exotic reversible phase transformation can significantly promote the formation of hierarchical nanolaminate dual‐phase structures that are self‐refining, thereby not only prominently enhancing work hardening and strengthening capacity but also providing an efficient pathway for microstructural modification to harvest optimized mechanical performance.

The exploration of bidirectional phase transformation in the MPEAs is still nascent but holds significant mechanistic intrigue in creating a step‐change in our current understanding of the dynamic structure‐property relationship. Moreover, comprehensively delving into the intrinsic nature of such captivating transformation reversibility has the potential to pave the way for a novel microstructure‐driven approach to alloy design. Herein, we aim to provide an updated review of this fascinating nanoscale reversible deformation process, focusing on its fundamental underlying mechanisms, physical origins, external influences, its impact on mechanical properties, and the outlook of future research. Our goal is to furnish a concise overview of groundbreaking discoveries and thought‐provoking concepts to stimulate a profound comprehension of crystal plasticity and inspire the advancement of innovative high‐performance alloys.

## 2 Underlying Mechanisms of Bidirectional Phase Transformation in MPEAs

Within the realm of shape memory alloys, reversible phase transformation triggered by temperature changes or reverse loading directions is not uncommon.^[^ ^57^ ^]^ However, the scenario of bidirectional phase transformation within MPEAs presents a distinct contrast. The first evidence of deformation‐induced bidirectional transformation was reported in the metastable Fe_50_Mn_30_Co_10_Cr_10_ MPEA under uniaxial loading.^[^ ^45^ ^]^ This unanticipated deformation process, involving a forward martensitic transformation from a metastable FCC structure to the HCP phase succeeded by a backward transformation from the HCP phase to FCC/FCC twinned structures, has since been documented in a growing body of experimental studies in various MPEAs.

**Figure 1:**
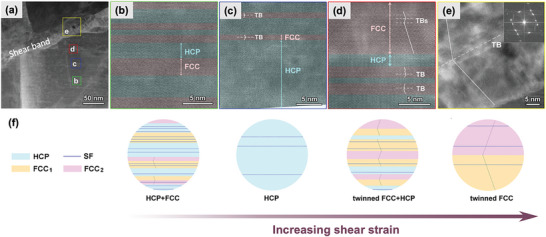
Activation of the bidirectional phase transformation and nanostructural evolution during in‐situ micropillar compression testing of a hierarchically nanostructured CoCrNi MPEA containing a mixture of FCC and HCP phases.^[^ ^48^ ^]^

As demonstrated in **Figure** **1**, bidirectional phase transformation induced plasticity was activated during in‐situ micropillar compression testing of a hierarchically nanostructured CoCrNi MPEA containing a mixture of FCC and HCP phases.^[^ ^47^, ^48^ ^]^ Owing to the high nonequilibrium processing nature of magnetron sputtering, the dual‐phase nanostructure of CoCrNi MPEA is notably different from those of coarse‐grained single‐phase FCC counterparts produced through conventional metallurgical approaches.^[^ ^41^, ^43^, ^47^, ^48^ ^]^ Such characteristic microstructures endow us with an incomparable opportunity to trace the dynamic process of dual phase transformation, which is manifest in the mechanically‐driven microstructural evolution. Figure 1a reveals that the plastic deformation of CoCrNi MPEA micropillars is primarily accommodated by the activation of a highly localized shear band. Within the rectangles marked b, c, d, and e, the enclosed regions undergo varying degrees of local shear strains that intensify from the periphery toward the center of the shear band.^[^ ^48^ ^]^ Regions distant from the shear band (Figure 1b) maintain the original mixture of HCP and FCC phases with the existence of twin boundaries (TBs) and stacking faults (SFs). Closer to the shear band, where larger shear strains are experienced (Figure 1c), a predominant HCP structure with a few nanotwinned FCC laths is the typical nanostructural trait, indicating the emergence of FCC to HCP phase transformation with increasing strain. Notably, in the vicinity of the shear band (Figure 1d), a profusion of twinned FCC structures alongside minimal HCP phases is evident, signaling the reverse transformation from the HCP phase to nanotwinned FCC structures upon further deformation. Within the shear band itself (Figure 1e), a single‐phase FCC twinned structure with a common {111}_FCC_ twin plane can be verified by the atomic structure and FFT pattern (inset). As schematically illustrated in Figure 1f, the dynamic progress of bidirectional phase transformation is achieved through a startling pathway of FCC → HCP → nanotwinned FCC as shear deformation increases, signifying a new twinning mechanism referred to as transformation‐mediated twinning (TMT).^[^ ^55^, ^56^ ^]^

**Figure 2:**
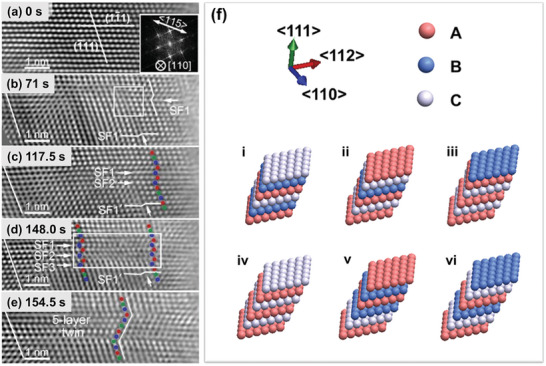
a–e) Real‐time atomic‐scale observation of reversible phase transformation assisted twinning.^[^ ^52^ ^]^ f) Schematic illustration of the atomistic pathway of bidirectional phase transformations.

Electron diffraction and atomistic characterization have substantiated that the orientation relationship between FCC and HCP is the well‐known Shoji‐Nishiyama (S‐N) relationship, i.e., {111}_FCC_∥(0001)_HCP_ and ${\left\langle 110\right\rangle }_{\mathrm{FCC}}∥{\left\langle 11\bar{2}0\right\rangle }_{\mathrm{HCP}}.$ ^[^ ^42^, ^45^, ^48^ ^]^ From the crystallographic perspective, the movement of Shockley partials creates stacking faults, which are 2D defects interrupting the regular periodic arrangement of atom layers in crystalline solids. When stacking faults occur, the stacking sequence of …ABCABCABC…. in FCC phases becomes …ABCBCACABC…. Therefore, the formation of stacking faults could be a prerequisite for the initiation of phase transformation. Considering the lattice geometry of FCC and HCP structures, it is plausible to infer that the mechanistic origin of the dual phase transformation is the facile slip of partial dislocations on the close‐packed {111}_FCC_∥(0001)_HCP_ planes in both FCC and HCP phases.^[^ ^48^ ^]^ Explicitly, the FCC‐to‐HCP transformation proceeds via the easy glid of Shockley partials with the Burgers vector of $\frac{1}{6}{\left\langle 112\right\rangle }_{FCC}$ on every other close‐packed {111}_FCC_ plane, altering the FCC stacking sequence of …*ABCABC*… to an HCP stacking sequence of …*ACAC*…. Conversely, the reversible HCP → FCC transformation is enabled by the gliding of Shockley partials with Burgers vectors $\frac{1}{3}\left\langle 1\bar{1}00\right\rangle$ on alternate {0002} basal plane.^[^ ^45^, ^46^, ^47^, ^48^, ^49^, ^50^, ^51^, ^52^, ^53^, ^54^, ^55^, ^56^ ^]^ By recourse to the in situ nano‐tensile testing on the single‐crystal CoCrNi MPEA with a single FCC structure, as demonstrated in **Figure** **2**a–e, this atomistic pathway of bidirectional phase transformation and TMT via the cooperative slip of partial dislocations has been captured and unraveled through the real‐time observations of deformation‐induced nanostructural evolution at the atomic scale.^[^ ^52^ ^]^ In essence, the panoramic process of FCC → HCP → nanotwinned FCC can be summarized schematically in Figure 2f. Theoretical investigations suggest that although the transformation of FCC →  HCP is readily activated, the continuous growth of the HCP phase is restricted by the kinetic barrier.^[^ ^38^, ^39^ ^]^ Nevertheless, the shear instability of the basal plane of the HCP lattice promotes the formation of FCC nanotwins,^[^ ^48^, ^55^ ^]^ charting a new twinning trajectory that is fundamentally varying from the classic scripture of twin nucleation through the layer‐by‐layer emission of Shockley partial dislocations on consecutive close‐packed planes.^[^ ^55^, ^56^, ^58^, ^59^, ^60^ ^]^

**Figure 3:**
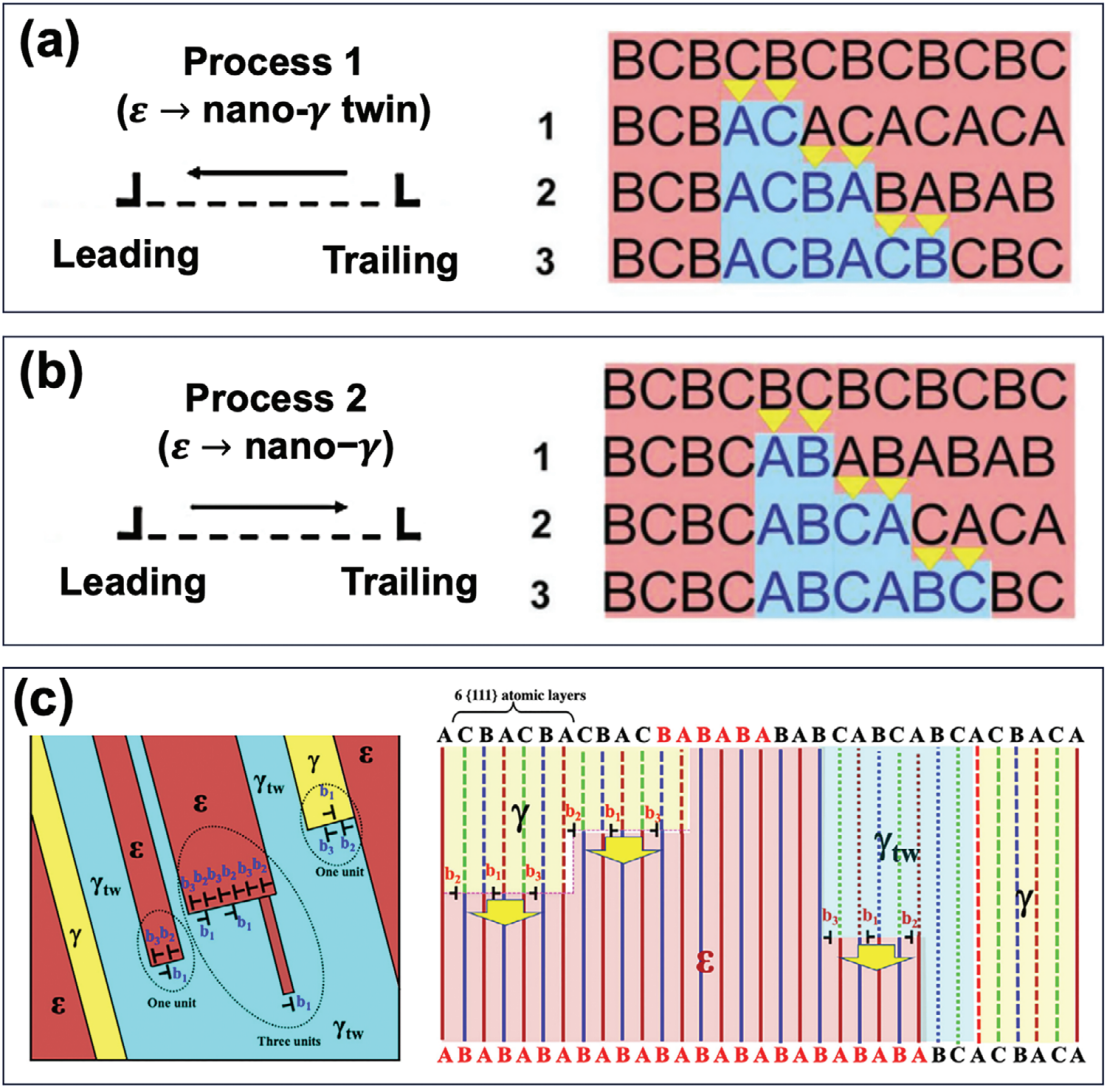
a) Schematic demonstrating the reverse transformation enabled by trailing Shockley partial dislocations, leading to transformation‐mediated twinning.^[^ ^45^ ^]^ b) Schematic showcasing the reverse transformation enabled by leading Shockley partials, forming the FCC matrix.^[^ ^45^ ^]^ c) Schematic illustration of the reverse transformation process dominated by an array of Shockley partial dislocations, represented by three partials with Burgers vectors of b_1_, b_2_, and b_3_.^[^ ^50^ ^]^

The dynamic bidirectional phase transformation is instrumental in unravelling the complex interplay between the new HCP phase, derived from the FCC structure, and its contribution to plasticity. While basal, non‐basal slips, and $\left\{10\bar{1}1\right\}$ twinning are engaged to facilitate deformation along the <a> and <c> crystallographic axes, the reverse transformation from the intermediate HCP phase to the FCC structure serves as a pivotal micromechanical mechanism.^[^ ^50^ ^]^ The activation of $\left\{10\bar{1}1\right\}$ twinning activities has been notably influential in facilitating the HCP →  FCC shear transformation.^[^ ^50^ ^]^ This transformation reversion regulates the nanoscale deformation process, reconfigures the lattice defects, and promotes strain delocalization.^[^ ^61^ ^]^ In theory, as an energetically favorable process, a full dislocation can congenitally dissociate into a leading partial dislocation and a trailing one to minimize their energies.^[^ ^62^ ^]^ Hence, the reverse HCP →  FCC transformation could be propelled by the movement of trailing or leading partial dislocations, with the selection dependent on their respective Schmid factors. This will create two distinct microstructural scenarios in terms of nanotwinned FCC or FCC structures, as depicted in **Figure** **3**a,b, respectively.^[^ ^45^ ^]^ In principle, when the Schmid factor of the trailing partial prevails over that of the leading one, the formation of the nanotwinned FCC structures that are transformed from the HCP lattice can be robustly accomplished via process 1 (Figure 3a). As demonstrated, the gliding of trailing partial dislocations on every second basal plane will transform the HCP phase into an FCC variant with a stacking sequence of …*CBACBA*…, exhibiting a twin relationship with the original …*ABCABC*… FCC matrix. In contrast, process 2, as illustrated in Figure 3b, is initiated and dominated by the motion of leading partials with a higher Schmid factor, reverting the HCP back to the original FCC structure.^[^ ^45^, ^47^, ^50^ ^]^ Therefore, no matter of forward or backward phase transformation, it is driven by the successive gliding of Shockley partials (leading or trailing) on every two close‐packed planes.

Beyond the abovementioned overriding mechanism, the collective gliding of three Shockley partial dislocations with Burgers vectors b_1_, b_2_, b_3_ on every two basal planes, amounting to six atomic planes in a unit, can catalyze the reverse HCP →  FCC/FCC nanotwin transformation, which is intimately associated with the configuration characteristics of three Shockley partials, as described in Figure 3c.^[^ ^50^ ^]^ Topologically, this repeatable array of Shockley partial units closely resembles the dislocation structures of Σ3 {112} incoherent TBs in FCC metals, where the movement directions determine the activation of twinning or detwinning processes.^[^ ^63^, ^64^, ^65^, ^66^, ^67^ ^]^ Notably, the sum of the Burgers vectors of the partials in one unit equals zero.^[^ ^63^ ^]^ Although prior research proposed that shear stress can effectively prompt the collective glide of the partial unit in FCC metals, further investigations are required to unravel the detailed underlying mechanisms underpinning this new path of reverse phase transformation.^[^ ^64^, ^67^ ^]^ In short, the adaptable stacking sequences of those close‐packed atomic layers play overarching roles in dual phase transformation.^[^ ^48^ ^]^ However, deformation‐driven phase transformation generally adheres to the principle of the minimization of Gibbs free energy, where a metastable phase tends to transform to a stable phase under external stimulus. This makes the occurrence of bidirectional phase transformation somewhat counterintuitive. The subsequent section will delve into the intrinsic material parameters and external influencing factors that crucially promote the distinctive FCC → HCP → FCC/nanotwinned FCC transformation.

## 3 Intrinsic and External Factors Affecting the Bidirectional Transformation Induced Plasticity (B‐TRIP)

As well recognized, the mechanics of crystal plasticity are fundamentally governed by the elemental deformation mechanisms, including dislocation slip, deformation twinning, and phase transformation.^[^ ^68^ ^]^ The initiation of these micromechanical processes is primarily regulated by intrinsic material parameters such as crystal structure, SFE, and crystallographic orientation. In addition, given the thermally activated nature of plastic deformation,^[^ ^69^ ^]^ the external loading ingredients such as temperature, strain rate, and pressure, would vitally fashion dislocation dynamics. To acquire a comprehensive description of the B‐TRIP effect, we herein critically examine the effects of SFE and external factors that boost the deformation‐driven transformation reversibility in MPEAs.

### 3.1 The Critical Role of Intrinsic Stacking Fault Energy

In materials with close‐packed crystal structures, intrinsic SFE (γ_isf_) is the most paramount parameter that essentially dictates the dissociation of a full dislocation into two partial dislocations separated by a stacking fault with a certain width. This separation is integral to the formation of distinct dislocation substructures, twinning activities, and phase transformation.^[^ ^70^ ^]^ Classic dislocation theory illuminates that the SFE represents the excess crystal energy per unit area of stacking fault.^[^ ^70^ ^]^ Therefore, the higher the SFE, the smaller the separation between the partial dislocations and the thinner the stacking fault will be, and vice versa. In materials with high and medium SFEs, dislocation slip typically dominates the plastic deformation, whereas materials with low SFEs tend to initiate deformation twinning that sculpts their internal microstructures.^[^ ^71^, ^72^, ^73^ ^]^ In the metastable austenitic steels and Fe‐Mn steels, the lower SFE will significantly boost the mechanically driven phase transformations.^[^ ^74^ ^]^ Consequently, the intrinsic SFE serves as a reliable indicator of the activation of the fundamental microscopic mechanisms within an alloy system, forecasting the momentous significance of precisely evaluating the SFE.

**Figure 4:**
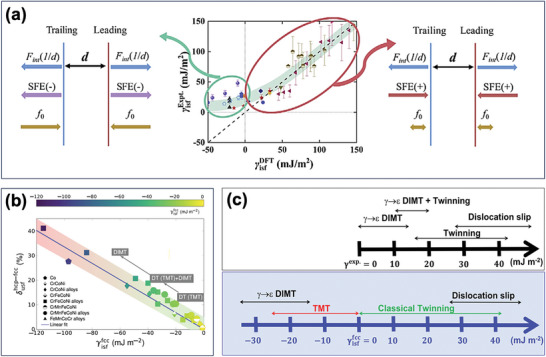
a) Correlation between theoretical SFE and experimentally measured SFE (middle image) and Schematic of the force balance for partial dislocations with positive SFE (right) and with negative SFE (left), corresponding to the red and green ellipse, respectively, marked in the middle image.^[^ ^75^ ^]^ b) Relationship between theoretical SFE and $\delta_{usf}^{hcp-fcc}$ in various metastable MPEAs, demonstrating the significance of two parameters in governing the microscopic deformation mechanisms.^[^ ^56^ ^]^ c) Divergence between the experimental SFE‐deformation mechanism relationship (top) and the theoretical SFE‐deformation mechanism relationship (bottom).^[^ ^56^ ^]^

The experimental measurement of SFE mainly relies on meticulously fathoming the equilibrium separation distance (*d*) between the partial dislocations,^[^ ^70^ ^]^ which is defined by the balance between the repulsive force stemming from the elastic interaction between two partials and the attractive force arising from the SFE, as depicted in the right part of **Figure** **4**a.^[^ ^75^ ^]^ Besides, ab initio calculations based on density functional theory are also a principal approach to accurately determining the SFEs.^[^ ^32^, ^39^, ^40^, ^75^ ^]^ For the conventional metals and alloys, there is a satisfactory agreement in composition‐dependent SFE between experimental and ab initio results, as indicated by the red ellipse in the middle image of Figure 4a.^[^ ^75^ ^]^ However, when tackling the new material paradigm of MPEAs, theoretical calculations often yield negative SFE values, in excellent alignment with the negative Gibbs energy difference between the HCP (ε) and FCC (γ) phases (Δ*G* ^γ → ε^), manifesting the thermodynamical metastability of these FCC structures.^[^ ^32^, ^39^, ^40^, ^75^ ^]^ The implication of the negative SFE is that the SFE and the elastic repulsive force bear the same direction, which should generate infinite partial separation. This utterly contradicts experimental observations that underpin the positive SFEs, as marked by the green ellipse in Figure 4a.^[^ ^75^ ^]^ Although the statistical fluctuations in compositional and packing arrangements of the various elements promote the occurrence of local chemical order (LCO) and create a wide spectrum of “local” SFEs ranging from positive to negative values,^[^ ^32^, ^76^ ^]^ this does not readily interpret the conflict between the experimental positive and theoretical negative SFEs in these MPEAs. To address this conundrum, the lattice friction force on the partial dislocations has recently been considered to critically ensure the force balance conditions and cement the finite partial separation, as schematically illustrated in the left image of Figure 4a.^[^ ^75^ ^]^ In conventional FCC metals and alloys, the Peierls‐Nabarro stress arising from the periodicity of the crystal lattice is typically marginal.^[^ ^70^ ^]^ Comparatively, in MPEAs, the high lattice distortion, coupled with the chemically complex environment consequentially, consequently heightens the elemental lattice barrier, thereby causing large lattice friction stress that restricts dislocation movement.^[^ ^13^, ^75^, ^77^ ^]^ In fact, by judiciously selecting the alloying elements to enhance atomic size differences and fluctuations in atomic bonding, significant lattice distortion can be achieved and utilized to strengthen the MPEAs, such as in VCoNi,^[^ ^78^ ^]^ where the lattice friction of dislocation motion is extraordinarily large. In light of the breakdown of the equilibrium between the SFE and the elastic repulsive force, obtaining experimental measurements of SFE in these metastable alloys poses a remarkable challenge. Nevertheless, theoretical evaluations of the SFE could be a rigorous beacon to elucidate the physical mechanisms. Such theoretical perspectives are invaluable for enriching and expanding our mechanistic understanding of the intricate structure‐property relationships inherent to these metastable systems.

The bidirectional phase transformation, which encompasses the forward FCC →  HCP transformation and the backward HCP →  FCC/FCC nanotwin transformation, is mechanistically rooted in the facile slip of Shockley partials between the close‐packed {111}_FCC_∥(0001)_HCP_ atomic planes in both FCC and HCP phases.^[^ ^48^ ^]^ This unique transformation capability arises from the flexible stacking sequence of those close‐packed atomic layers, which is underlay by the negative theoretical SFE and the thermodynamic instability of the metastable FCC phase.^[^ ^56^ ^]^ In the HCP structure, the primary basal slip is prevalent and more readily activated compared to other deformation mechanisms. In a previous theoretical investigation, researchers introduced the concept of the relative effective energy barrier, denoted as $\delta_{usf}^{hcp-fcc}=\;\left(\gamma_{usf}^{hcp}-\gamma_{usf}^{fcc}\right)/\gamma_{usf}^{fcc}$, defined as the difference in the unstable SFE of HCP and FCC phases relative to the unstable SFE of the FCC phase ($\gamma_{usf}^{hcp}$ and $\gamma_{usf}^{fcc}$ represent the energy barriers for creating a stacking fault in the close‐packed crystal planes of the HCP and FCC structures, respectively).^[^ ^56^ ^]^ This physical parameter is crucial for quantifying the compatibility for activation of partials sequentially in the initial FCC matrix and in the newly formed HCP phase. A small $\delta_{usf}^{hcp-fcc}$ signifies the comparable energy barriers of SF formation in both HCP and FCC structures.^[^ ^56^ ^]^ As exhibited in Figure 4b, the $\delta_{usf}^{hcp-fcc}$ increases apparently with the decrease of the $\gamma_{isf}^{fcc}$ in an approximately linear manner. Therefore, reducing the negative $\gamma_{isf}^{fcc}$ can provide a significant driving force to make the FCC →  HCP transformation more thermodynamically favorable. However, an excessively negative $\gamma_{isf}^{fcc}$ can severely inhibit the reverse transformation because of the higher SFE of the HCP phase, hampering the dissociation of a full dislocation on the basal planes. Consequently, a spectrum of small negative $\gamma_{isf}^{fcc}$ along with small $\delta_{usf}^{hcp-fcc}$ are the most pivotal material parameters for enabling the dual phase transformation and TMT.^[^ ^56^ ^]^ For instance, the $\gamma_{isf}^{fcc}$ and $\delta_{usf}^{hcp-fcc}$ of CoCrNi MPEA are −21 mJ m^−2^ and 6.2%, respectively, while those values of Fe_50_Mn_30_Co_10_Cr_10_ are −21 mJ m^−2^ and 10.8%, respectively.^[^ ^56^ ^]^ As illustrated in Figure 4b, as the theoretical negative $\gamma_{isf}^{fcc}$ decreases, the overarching deformation mechanisms can be shifted from the traditional deformation twinning (DT) and TMT to the synergetic activation of TMT and deformation‐induced martensitic transformation (DIMT), ultimately leading to DIMT with the stable HCP phase as the final nanostructures.^[^ ^56^ ^]^ Despite the intricate chemical complexities of MPEAs, the theoretical $\gamma_{isf}^{fcc}$ can be a reliable predictor for the dominant micromechanical mechanisms. Furthermore, in comparison to the relationship between the deformation mechanisms and experimentally measured SFE, as depicted in Figure 4c, theoretical SFE, derived from calculating the intrinsic energy barriers, renders a better resolution in discerning whether the full or partial‐mediated plasticity is operational, broadening the horizon of our understanding of fundamental deformation physics.^[^ ^56^ ^]^

In addition, it is vital to highlight that the range of $\gamma_{isf}^{fcc}$ value for initiating bidirectional phase transformation and TMT spans from −10 to −50 mJ m^−2^.^[^ ^56^ ^]^ On account of the immanent dependence of the SFE on the chemical composition, this critical insight allows for elaborate adjustment of alloy metastability and deformation‐driven transformation reversibility. In a nutshell, the negative SFE signals the novel dislocation mechanisms and lays the relevant foundation to streamline the screening process for the mechanistic design of advanced MPEAs with vast compositional space.

### 3.2 Effects of External Loading Conditions

Dislocations unremittingly encounter obstacles as they navigate through the crystal lattice.^[^ ^68^, ^69^, ^70^ ^]^ The short‐range interactions between dislocations and energy barriers occur in localized regions containing hundreds or thousands of atoms, where thermal vibrations are essential.^[^ ^69^ ^]^ Classical crystal plasticity theory enlightens that the microscopic processes of dislocation dynamics are thermally activated events at the atomic scale.^[^ ^69^, ^70^ ^]^ This fundamental perception of deformation physics posits that thermal activation can assist dislocations in surmounting the energy barriers that dictate their movements.^[^ ^69^ ^]^ This cardinal principle affirms that, beyond the SFE and other intrinsic material properties, elemental dislocation mechanisms are, to some extent, critically pertinent to the external loading conditions, particularly temperature and strain rate. Taking pure Cu for example, extensive full dislocation activities are the primary carrier of plastic deformation at room temperatures and low strain rate, whereas lowering the temperatures and enhancing loading rates tend to constrict the dislocation motion and foster deformation twinning that considerably accommodates plasticity.^[^ ^9^, ^79^ ^]^ Notably, in FeMnCoCrNi‐based MPEAs, given the HCP structure is more thermodynamically favorable at low temperatures due to its lower energy than its FCC counterpart, the cryogenic‐deformation‐induced phase transformation has been well documented.^[^ ^42^, ^43^ ^]^ In the case of metastable Fe_50_Mn_30_Co_10_Cr_10_ MPEA, it was proposed that reverse HCP →  FCC/FCC nanotwin transformation was kinetically instigated by local dissipative heating and local stress/strain fields.^[^ ^45^ ^]^ These studies illuminate the complex interplay between thermal activation and dislocation mechanics. Herein, we showcase a selection of groundbreaking studies to underscore the profound impact of deformation conditions on the activation of the B‐TRIP effect.

**Figure 5:**
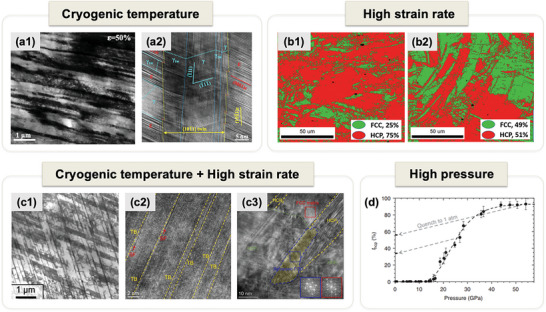
Effects of external loading conditions on the B‐TRIP effect. *Effects of cryogenic temperatures*:^[^ ^50^ ^]^ a1) Dislocation substructures of Cr_26_Mn_20_Fe_20_Co_20_Ni_14_ MPEA deformed at room temperature, a2) Activation of bidirectional phase transformation at 4.2K. *Effects of high strain rate loading*:^[^ ^51^ ^]^ b1) Phase mapping of Fe_50_Mn_30_Co_10_Cr_10_ MPEA after quasistatic compression, b2) Phase mapping of Fe_50_Mn_30_Co_10_Cr_10_ MPEA after dynamic compression with a strain rate of 3200 s^−1^. *Combined effects of cryogenic temperature and high strain rate*: c1) Microstructures of CoCrNi MPEA deformed at 77 K,^[^ ^41^ ^]^ c2) Formation of nanotwins and SFs in CoCrNi MPEA after dynamic compression at room temperature,^[^ ^53^ ^]^ c3) Activation of B‐TRIP mechanism in CoCrNi MPEA after dynamic compression at 77 K.^[^ ^53^ ^]^ *Effects of high pressure*.d) The HCP phase fraction of CrMnFeCoNi as a function of pressure.^[^ ^82^ ^]^

Extensive theoretical investigations have elucidated the temperature‐dependence of SFE in FeMnCoCrNi‐based MPEAs, verifying that their SFE diminishes as temperature decreases, which in turn directly influences deformation mechanisms.^[^ ^80^ ^]^ As demonstrated in **Figure** **5**a1, at room temperature, the plastic deformation of the Cr_26_Mn_20_Fe_20_Co_20_Ni_14_ MPEA is predominantly governed by full dislocation activities, which eventually evolve into dislocation walls as deformation progresses. Upon deformation at 77 K, the primary deformation modes shift toward deformation twinning and the FCC →  HCP phase transformation.^[^ ^50^ ^]^ Further decreasing the temperature to 4.2 K reveals a more diverse and complex microstructural response, highlighting the intricate nature of the microscopic deformation processes under extreme cryogenic conditions. In addition to the substantial FCC →  HCP transformation, $\left\{10\bar{1}1\right\}$ twinning within the HCP phase can be activated. This type of twinning, capable of accommodating deformation along the <c> axis in the HCP structure, greatly facilitates the HCP → nanotwinned FCC/FCC reverse transformation, both within and in the vicinity of the twin bands, as illustrated in Figure 5a2.^[^ ^50^ ^]^ Moreover, the scenarios describing the transformation reversion (Figure 3c) can also be detected. Therefore, the reduction of the SFE in a cryogenic environment supplies abundant thermodynamic driving force to propel the FCC →  HCP transformation. Concurrently, the local dissipative heating and complex stress/strain fields provide the kinetic energy necessitated to drive the dynamically reverse transformation. This reversion is facilitated by the small difference in cohesive energy between two phases at low temperatures, allowing for easier transition from one to another under the proper conditions.^[^ ^50^ ^]^ This interplay between thermodynamic and kinetic factors at cryogenic temperatures is critical for managing phase transformations in MPEAs. These findings are instrumental in understanding and predicting the mechanical behavior of MPEAs under different temperature regimes, particularly for applications that require robust performance at cryogenic temperatures.^[^ ^81^ ^]^

Apart from reducing the temperatures, enhancing the strain rate can have a significant impact on the deformation behavior of metals and alloys. Unlike the heightened tendency for deformation twinning triggered by high‐strain‐rate deformation, the accompanying adiabatic heating can elicit complex effects on the energetics of the transformation process. Consider the dual‐phase Fe_50_Mn_30_Co_10_Cr_10_ MPEA comprising 55% FCC and 45% HCP, as an example.^[^ ^51^ ^]^ Under quasistatic deformation conditions, there is a bidirectional phase transformation, but the final microstructural fingerprint is the dominant HCP structure with a minor presence of the FCC phase (Figure 5b1), suggesting a notable forward transformation after 37% compression. In contrast, when subjected to dynamic compression at a strain rate of 3200 s^−1^ and a strain of 48%, the volume fraction of the HCP phase marginally increased to 61%.^[^ ^51^ ^]^ In‐depth microstructural characterization and constitutive analysis have revealed that adiabatic heat generated during high‐strain‐rate deformation can actively impel the displacive reverse transformation and enhance FCC phase stability, thereby aiding in the preservation of the FCC structure fraction. The unique B‐TRIP and heterogeneous deformation mechanisms play pivotal roles in averting stress/strain concentrations and endowing this MPEA with enhanced deformability under high strain rate conditions.^[^ ^51^ ^]^ These insights are not only advantageous for enriching our understanding of how MPEAs respond to dynamic deformation but also crucial for improving material design to withstand sudden and extreme loading conditions in various fields such as impact engineering, ballistics, and automotive industries. Additionally, more extreme loading conditions that combine the cryogenic temperatures with high strain rates can trigger the B‐TRIP mechanism in the coarse‐grained CoCrNi MPEA, as presented in Figure 5c3, which cannot be achieved solely through the deformation at low temperatures (Figure 5c1) or under dynamic deformation (Figure 5c2).^[^ ^53^ ^]^ Furthermore, while the categorization of high pressure‐induced transformations as bidirectional may not be strictly accurate, as revealed in Figure 5d,^[^ ^82^ ^]^ it is noteworthy that in the Cantor alloy, the potential for forward FCC →  HCP transformation is awakened under high pressure, while reverse HCP‐to‐FCC transformation takes place upon pressure releases, underscoring the metastable nature of the HCP phase in this alloy.^[^ ^82^, ^83^ ^]^

Fundamentally, external loading conditions furnish necessary thermodynamical and/or kinetical tractive forces that are essential for initiating the B‐TRIP effect in MPEAs. It is crucial to realize that the intrinsic SFE of these MPEAs must closely align with the SFE spectrum that favors dual‐phase transformations; significant deviation from this range could render the alloys' micro‐plasticity mechanisms less sensitive to external stress inputs.^[^ ^9^, ^75^ ^]^ Overall, a thorough comprehension of how the external loading influences the B‐TRIP effect can profoundly advance and deepen our knowledge of this unique mechanistic phenomenon, facilitating the establishment of accurate and predictive models of material behavior. More significantly, it paves an innovative avenue for groundbreaking microstructural design toward exceptional mechanical properties to meet the demands of critical applications.

## 4 Excellent Mechanical Properties of MPEAs Enabled by B‐TRIP Effect

Strengthening materials traditionally involves managing the creation of internal defects and incoherent boundaries to obstruct dislocation motion. However, these strategies invariably sacrifice the work‐hardening capacity of materials and then compromise their ability to accommodate plastic deformation, leading to the notoriously known strength‐ductility trade‐off.^[^ ^3^ ^]^ In stark contrast, micromechanical mechanisms such as TWIP and TRIP can undertake substantial plasticity and generate high‐density nanoscale cTBsc or phase boundaries (PBs). These boundaries act as effective barriers to dislocation motion, yielding a dynamic “Hall‐Petch” effect, and facilitate the accumulation of additional defects, empowering the accomplishment of high strength, considerable work‐hardening capacity, and exceptional ductility.^[^ ^61^ ^]^ These forward‐thinking strategies are beaconed by the fundamental mechanistic principle of introducing extra strengthening mechanisms and enabling a spread‐out distribution of plastic flow to achieve strain delocalization and postpone the onset of premature plastic instability.^[^ ^34^ ^]^

Since the microscopic plastic mechanisms are at the heart of the manifestation of global mechanical properties, it is rational to anticipate that bidirectional phase transformation is pivotal in augmenting the mechanical performance of MEPAs. From a mechanistic standpoint, the continuous gliding of Shockley partial dislocations on every second {111} atomic plane facilitates the forward FCC →  HCP phase transformation, generating a shear strain of 35%.^[^ ^48^ ^]^ The intermediate HCP phase can transition back to the FCC structure via the motion of Shockley partials with Burgers vectors $\frac{1}{3}\left\langle 1\bar{1}00\right\rangle$ on alternate {0002} basal plane, inducing an additional shear strain of 35%.^[^ ^48^ ^]^ This transformation reversion enables a cumulative increase of shear strain in the same direction as the preceding phase transformation, ultimately achieving an extensive shear with a total shear transformation strain of 70% throughout the bidirectional phase transformation process.^[^ ^48^ ^]^

**Figure 6:**
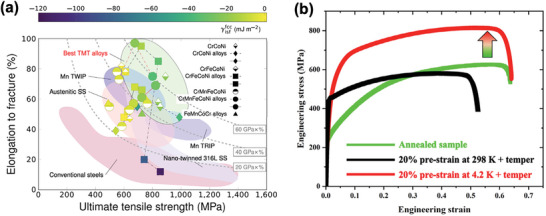
a) Plot of ultimate tensile strength against elongation of fracture for a wide range of metals and alloys, showcasing the exceptional combination of high strength and excellent ductility in intrinsically metastable MPEAs enabled by the B‐TRIP effect.^[^ ^56^ ^]^ b) Mechanical performance of Cr_26_Mn_20_Fe_20_Co_20_Ni_14_ MPEA after different processing, demonstrating the mechanical merits of the cutting‐edge NL‐DP nanostructures stemming from the bidirectional phase transformation.^[^ ^50^ ^]^

In conventional TRIP alloys, dislocation motion is significantly constrained by PBs, leading to the accumulation of dislocations and thus large local stress concentrations, which cause high susceptibility to internal damage initiation. Microcracks tend to nucleate and propagate from these strong interfaces due to the considerable local mechanical disparities between the constituting FCC and HCP phases. In contrast, the deformation‐driven transformation reversibility can be identified as a vital self‐adjusting micromechanical mechanism.^[^ ^45^, ^47^, ^50^, ^51^ ^]^ It can reorganize and redistribute defect arrangement and stress/strain fields, effectively mitigating the intense local stress that is the primary culprit of damage initiation. At the microstructural level, the hierarchical nano‐laminated dual‐phase (NL‐DP) structures, encompassing FCC matrix, HCP nanolaminate, and FCC nanotwins, are established through the dynamic interplay of the forward and reverse phase transformation.^[^ ^45^, ^50^ ^]^ These nanoscale soft FCC phase and hard HCP structure can coordinate a synergistic activation of various strengthening and toughening mechanisms. As this mechanistic process unfolds, the hierarchical nanolaminate structure is continuously self‐refining, tremendously curtailing the mean free path for dislocation motion and thereby achieving the dynamic Hall‐Petch effect. In addition, the profuse TBs and PBs play essential roles in facilitating dislocation storage and impeding dislocation annihilation to suppress dynamic recovery.^[^ ^84^, ^85^ ^]^ By enhancing the hardening component and limiting the softening component, the work‐hardening capacity of the alloys can be boosted prominently to delay the onset of localized deformation.^[^ ^68^, ^71^ ^]^ These features collectively promote substantial strengthening while preserving ductility. Therefore, as depicted in **Figure** **6a**,^[^ ^56^ ^]^ the “best TMT” alloys with a high propensity for B‐TRIP demonstrate an exceptional combination of strength and ductility, offering great potential to overcome the dilemma of strength‐ductility trade‐off. The virtually limitless compositional space of MPEAs equips us with a potent capacity to judiciously design alloys that leverage the benefits of the B‐TRIP effect, forging a new frontier in the development of high‐performing alloys previously unattainable.

Expanding upon the mechanical merits offered by this distinguished physical mechanism, the hierarchical NL‐DP structures empower us to precisely customize the microstructures of MPEAs to optimize their mechanical properties. As illustrated in Figure 6b,^[^ ^50^ ^]^ the Cr_26_Mn_20_Fe_20_Co_20_Ni_14_ MPEA, when subjected to pre‐straining at 4.2 K and followed by appropriate tempering, demonstrates a much‐enhanced strength without loss of ductility and work hardening capability. This improvement is in stark contrast to its annealed and cold‐worked counterparts, which exhibit either low or high dislocation densities. The key to this enhancement lies in the formation of the NL‐DP structures with plentiful interfaces. This significant microstructural length‐scale refinement can dramatically strengthen the alloy to a crucial level, endowing it with kinetical energy required to initiate a cascade of microscopic plasticity mechanisms, such as B‐TRIP, dislocation‐interface interactions, and twin‐twin interactions, which are unattainable in the annealed samples. Therefore, by adroitly manipulating loading and tempering conditions, we can precisely engineer the microstructures to induce the desirable NL‐DP structures, delivering critical perspectives for the microstructural design to underpin the simultaneous attainment of high strength and high ductility in advanced bulk nanostructured alloys.

## 5 Outlook and Perspective

**Figure 7:**
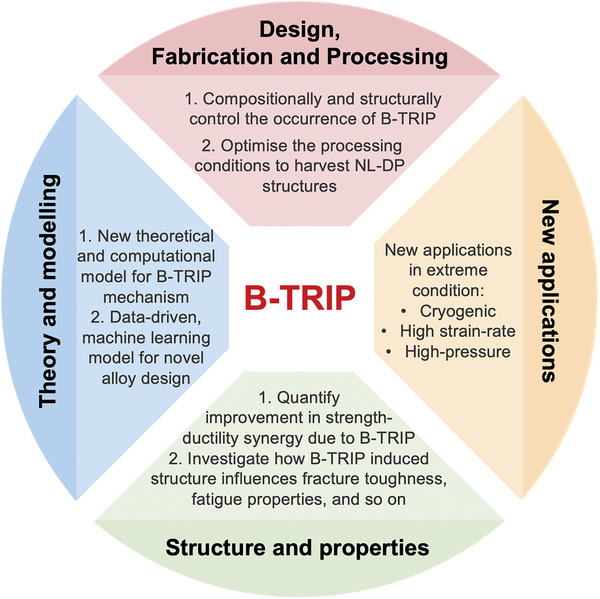
Open issues and challenges for elucidating and leveraging the B‐TRIP mechanism and the mechanistic design of next‐generation high‐performing MPEAs.

The emergence of MPEAs as a disruptive innovation in metallurgy, showcasing exceptional mechanical and functional properties, has sparked a surge of research interest. These innovative alloys, by virtue of their concentrated multi‐element composition, introduce substantial chemical heterogeneity and create an expansive compositional landscape, heralding a paradigmatic shift in the design of high‐performing alloys. Their ingrained chemical complexity confers them with unparalleled intrinsic material parameters and microscale plasticity mechanisms. In this concise review, we recapitulate the intrinsic theoretical negative SFE of FeMnCoCrNi‐based MPEAs, the unique bidirectional phase transformation, and its profound impact on mechanical properties. The B‐TRIP effect offers a persuasive interpretation for the interweaved coexistence of FCC nanotwins and HCP nanolamellae with a lateral thickness of several or tens of atomic layers, culminating in the NL‐DP composite structure. This startling microstructural phenomenon is not readily attainable through the TWIP or TRIP mechanisms. As evidenced in a growing body of research, the negative SFE furnishes the thermodynamic driving force for the forward FCC →  HCP transformation, while the kinetic energy, arising from localized dissipate heating, ultrahigh local stresses, and the intricate stress/strain field, facilitates the reverse HCP‐to‐FCC nanotwin/FCC transformation. The MPEA community and beyond have been increasingly recognizing the significance of this novel mechanistic pathway for its intrinsic ability to self‐accommodate and its critical roles in fostering massive plasticity alongside an exceptional synergy of high strength, large work‐hardening capacity, and exceptional ductility. As the relevant theoretical and experimental investigations continue to unfold in full swing, we highlight several critical uncharted issues and challenges, as illustrated in **Figure** **7**, to be addressed for advancing our understanding and harnessing the full potential of MPEAs in emerging applications.

The fundamental tenet in physical metallurgy is that a material's properties are primarily dictated by its chemical compositions and nano/micro‐structures. At a chemical level, strategic incorporation of alloying elements to subtly adjust the SFE of materials can delicately adjust their structural stability and micromechanical mechanisms, thereby initiating the beneficial B‐TRIP effect. From a structural perspective, optimizing fabrication and processing conditions to craft intriguing NL‐DP nanostructures is greatly advantageous for bolstering the mechanical performance of MPEAs. Despite significant progress made in enhancing the overall mechanical performance of metallic materials, there remains much room in the strength‐ductility/toughness space to expand into. Beyond intrinsic SFE, it is imperative to explore the significance of other material properties and microstructural characteristics in activating bidirectional phase transformation. These include shear/Young's modulus, chemical composition, grain size, and distribution,^[^ ^8^, ^86^ ^]^ dislocation substructures,^[^ ^87^ ^]^ second‐phase precipitation,^[^ ^88^ ^]^ interstitials,^[^ ^24^, ^89^ ^]^ degree of lattice distortion,^[^ ^78^ ^]^ and chemical short‐range order,^[^ ^15^, ^90^ ^]^ among others. The integration of additional innovations in microstructural engineering,^[^ ^91^, ^92^, ^93^, ^94^, ^95^, ^96^ ^]^ holds great potential to facilitate the synergistic activation and coordination of multiple strengthening and strain hardening mechanisms induced by local non‐homogeneous plastic deformation. This can be kinetically and dynamically alleviated by the B‐TRIP, yielding unprecedented mechanical properties beyond current benchmark ranges. Therefore, the correlative and coupling employment of high‐end manufacturing and processing techniques, together with meticulous control over these processes,^[^ ^97^, ^98^, ^99^, ^100^ ^]^ is indispensable for leveraging the mechanical advantages offered by B‐TRIP to forge next‐generation alloys that will likely redefine performance standards.

While the intricate atomistic dynamics of bidirectional phase transformation have been the subject of both theoretical and experimental scrutiny, a core scientific question pertinent to the exact contribution of the B‐TRIP phenomenon to the attendant mechanical properties remains elusive. The pivotal crux to elucidate this significant issue necessitates a quantitative assessment of how individual deformation events contribute to plasticity and properties, considering the complex hieratical mechanisms involved in B‐TRIP behavior. This highly unsatisfactory state in the structure‐property relationship hampers our ability to accurately predict mechanical behaviors based on microstructural characteristics. Furthermore, regarding the perspective engineering applications of advanced FeMnCoCrNi‐based MPEAs, a thorough understanding of their deformation responses to fracture, fatigue, and wear is essentially crucial owing to safety issues. Delving into the influence of the B‐TRIP effect on the fracture toughness, cyclic deformation properties, and wear resistance is critical for enhancing the alloy's durability and longevity and thereby promoting the sustainable development of high‐performance alloys.

Although the physical origin and atomistic pathways of B‐TRIP have been posited, the relationships among chemical composition, intrinsic SFE, nanostructure, and microscopic mechanisms of dislocation plasticity remain predominantly qualitative, rendering the compositional design largely empirical. Therefore, developing a holistic theoretical and computational model to quantitatively delineate the relationship of the composition‐SFE‐nanostructure‐deformation mechanisms is of paramount significance for establishing an integrated scientific framework for future mechanistic alloy design to expand the strength‐ductility nexus. Additionally, in light of the nearly infinite composition space of MPEAs, there is also a pressing need for the creation of robust data‐driven machine learning models.^[^ ^101^, ^102^ ^]^ Such models could profoundly expedite the design and development of innovative MPEA, streamlining the process of discovery and application. The newly developed MPEAs, which extend beyond FeMnCoCrNi‐based composition and feature the unique B‐TRIP effect, could deliver unprecedented combinations of strength, ductility, and toughness that were previously inaccessible. This positions them as ideal candidates for safety‐critical and load‐bearing structural applications. More significantly, these alloys can fulfill the increasingly stringent property demands for engineering applications and are adaptable to the toughest operative environments such as high impact, cryogenic temperatures, and high‐pressure conditions, common in industries like aerospace, deep‐sea exploration, and hydrogen sectors. This capability furnishes sufficient impetus and momentum to advance evolving technologies and catalyze the rising of new economies. All in all, the discovery and in‐depth exploration of the bidirectional phase transformation of MPEAs not only render us fresh insights that propel a quantum leap in understanding the deformation physics and metastability engineering of MPEAs, but also provide an innovative mechanistic design strategy for creating cutting‐edge alloys with superior mechanical properties. This pivotal breakthrough could stand as a significant milestone in the journey toward the next major technological frontiers.

## Conflict of Interest

The authors declare no conflict of interest.
